# Linking performance to powerhouse: mitochondrial aerobic metabolism in blood cells reflects flight endurance of house sparrows (*Passer domesticus*)

**DOI:** 10.1098/rsbl.2025.0199

**Published:** 2025-08-06

**Authors:** Riccardo Ton, Max M. Gillings, Hector Pacheco-Fuentes, Antoine Stier, Simon C. Griffith

**Affiliations:** ^1^ School of Natural Sciences, Macquarie University, Sydney, New South Wales, Australia; ^2^ Cavanilles Institute of Biodiversity, University of Valencia, Valencia, Valencian Community, Spain; ^3^ CNRS, Paris, France

**Keywords:** aerobic metabolism, ATP production, songbird, take off

## Abstract

Identifying the physiological mechanisms underpinning inter-individual differences in performance and fitness remains a key challenge in organismal biology. Variation in mitochondrial aerobic metabolism has been suggested to underlie inter-individual variation in performance, but this remains seldom tested, partly because of the need to use terminal sampling for assessing mitochondrial parameters. To fill this knowledge gap, we investigated whether inter-individual variation in mitochondrial aerobic parameters measured from less-invasively taken samples (i.e*.* blood cells) would correlate with both anaerobic and aerobic metrics of flight performance in house sparrows (*Passer domesticus*). We predicted that mitochondrial aerobic metabolism should correlate with aerobic but not anaerobic metrics of flight performance. As expected, we found no evidence for a relationship between mitochondrial metabolism and the energy required to take-off (i.e. anaerobic), but flight duration to exhaustion (i.e. aerobic) correlated positively with both cellular mitochondrial respiration rates and oxidative phosphorylation efficiency, a proxy of mitochondrial efficiency to convert nutrients into ATP. Our results, therefore, support the idea that inter-individual variation in mitochondrial aerobic metabolism could underlie variation in aerobic performance and suggest that the nucleated blood cells of birds (and potentially other non-mammalian vertebrates) may be a relevant biological sample to test those links.

## 1. Introduction

Understanding the physiological underpinnings of variation in organismal performance is a major goal of ecological and biomedical studies [1], but connecting different levels of organization can be challenging [2]. Mitochondrial aerobic metabolism is the subcellular process driving most energy transduction from nutrients to adenosine tri-phosphate (ATP), and recent studies have suggested that inter-individual differences in mitochondrial aerobic metabolism may explain variation in individual quality [3], immune function [4], social behaviour [5] and locomotion [6,7]. These studies highlight the great promise of mitochondrial metabolism as a metric of organismal performance, but further evidence is needed to ascertain these relationships [1].

The majority of measurements of mitochondrial aerobic metabolism are conducted using terminal tissue sampling, such as skeletal or cardiac muscles [8], brain [8,9] and liver [8–10]. However, in non-mammalian vertebrates, blood can also be used because red blood cells possess functional mitochondria [11–13]. This provides researchers with the opportunity to perform minimally invasive and longitudinal sampling [12], which is less disruptive than terminal sampling or tissue biopsies, especially in animals of small size [14]. However, the validity of blood cells as an appropriate substrate to obtain informative and biologically relevant mitochondrial measurements remains questionable. Blood cells are indeed responsible for minimal energy production [15] and therefore may not reflect whole-animal performance, although some evidence suggests that mitochondrial aerobic metabolism moderately correlates between blood cells and more active tissues such as skeletal muscle and brain [12,16].

In the last few years, evidence has started to indicate that mitochondrial metabolism measured in blood cells correlates with whole-animal resting metabolism [14,17], and that this relationship seems stronger than with other, metabolically more active, tissues such as brain, liver and muscles [15]. Given that whole-animal resting and maximum metabolism are considered heuristic metrics of organismal performance [18], mitochondrial metabolism in blood cells thus offers a valuable and relatively non-invasive approach for testing performance links between the mitochondrial and whole-organism levels. However, while the role of mitochondria in skeletal muscles is well known [19,20], whether blood cells metabolism correlates with ecologically relevant traits at the whole animal level remains untested to the best of our knowledge.

To explore this possibility, we estimated take-off energy and flight endurance in house sparrows, *Passer domesticus*, before measuring mitochondrial metabolism in their blood cells. Take-off energy expenditure and flight endurance are two traits with important fitness-related ramifications given their role in escaping predators, seeking food and locomotion [21]. However, while the former is an anaerobic process (an intense burst of energy within the first second of flight), the latter involves aerobic metabolism supported by the cellular energy provided by mitochondrial respiration [22]. Therefore, we predicted no relationship and a positive relationship between mitochondrial aerobic metabolism, take-off energy expenditure and flight endurance, respectively.

## 2. Material and methods

### (a) Animal model and housing conditions

We captured a total of 53 wild female adult house sparrows using mist nets in the three Australian cities/towns of Broken Hill (*n* = 34), Denman (*n* = 6) and Branxton (*n* = 13), in New South Wales, between 20 and 30 May 2023. We used adult females to avoid introducing variation in mitochondrial aerobic metabolism due to sex and age [23]. Groups of three females were housed together in 18 indoor cages 60 × 80 × 46 cm (height × width × depth). All the cages were in the same climate-controlled room kept at mean ± s.d. 23.34 ± 0.57°C and exposed to an 11 : 13 h cycle of light and darkness. Each cage contained at least one subject from each location when possible (Broken Hill, Denman and Branxton). The housing lasted for a total of 10 weeks, and birds were maintained with a dry seed finch mix plus chick starter pellets for domestic poultry and water ad libitum. While 10 weeks of habituation to captivity may plastically alter flight performances due to lower locomotion activity [20], recent evidence in a closely related species suggests otherwise [24]. Additionally, testing wild birds that have been brought to captivity very recently would have led to bias linked to stress since stress-related physiological metrics are known to be impacted for up to six weeks in wild house sparrows [25,26].

### (b) Take-off and flight endurance test

Two days before the beginning of mitochondrial measurements, we assayed anaerobic flight performance by building a fly-away structure as described by DiLiberto *et al*. [27]. We recorded each bird taking off from a perch on three GoPro Hero7 Black cameras (60 frames per second, 1440 pixels resolution) that were fixed in different positions to capture flight from different perspectives. We synchronized the video cameras with ambient sound and flashlight cues before recording a bout of take-off flights. Video recordings of each flight from each camera were analysed in Python v. 3.8.3 [28] using software Argus. After generating the three-dimensional coordinates in Argus, we computed the energy expended during take-off in joules [29]. Specifically, we calculated instantaneous velocity by subtracting the value of the previous frame’s (*n*−1) coordinates from the current (*n*). We multiplied the resultant vector magnitude by 60 (i.e. frame rate of recordings) to yield velocity (*v*) in m s^−1^ [30]. We used the resulting instantaneous velocity together with the mass of each bird (*m*), the vertical height achieved in each frame (*z*) and the gravity constant (*g*) to calculate both the instantaneous kinetic and potential energies. These were added to calculate the total energy expended in each frame [29]. The energy expended values over the 10 frames of take-off flight were averaged to produce mean energy expenditure during escape take-off flight. For further methodological details, see DiLiberto *et al.* [27].

To get an estimate of aerobic flight performance, we released individual sparrows into a row of eight connected cages with the dividers removed to form a tunnel shaped cage 60 × 400 × 46 cm (height × length × depth) and continuously chased the birds with an experimenter positioned at each end to elicit flight, from one end of the cage to the other. We timed flight endurance in seconds (s) and considered the measurements completed when the subject displayed clear signs of exhaustion such as panting, wing dropping and crouched posture, was incapable of additional flight and manually caught by hand. Our endurance test was elaborated as an alternative to the method used in previous studies where a motorized wheel elicited maximum peak locomotor activity [31]. During preliminary tests on different subjects, we found it hard to properly replicate this method with birds unwilling to move, getting extremely stressed, freezing and sliding along the bottom of the device. These unsuccessful attempts prompted us to apply the method presented in this study. While the reliability and reproducibility of our approach need to be validated against more standardized ways to measure flight endurance such as the use of wind tunnels [32], it represents an easy and accessible solution also to research laboratories with limited space or resources. These tests were performed prior to the mitochondrial measurements and were thus done completely blind to the subsequent data generated in those assays.

### (c) Mitochondrial measurements

Measurements of mitochondrial aerobic metabolism were performed over three weeks on intact blood cells using whole blood [33] and an O2k high-resolution respirometer (Oroboros Instruments, Innsbruck, Austria). The O2k was calibrated for background O_2_ consumption before the experiment and daily for 100% O_2_ levels. We collected 50 μl of blood from the brachial vein with a heparinized capillary and mixed it in an Eppendorf tube containing 0.95 ml MiR05 medium for mitochondrial respiration at 40°C (0.5 mM EGTA (ethylene glycol-bis(β-aminoethyl ether)-N,N,N',N'-tetraacetic acid), 3 mM MgCl_2_, 60 mM K-lactobionate, 20 mM taurine, 10 mM KH_2_PO_4_, 20 mM Hepes, 110 mM sucrose, free fatty acid bovine serum albumin (1 g l^−1^) and pH 7.1). The blood suspension was gently mixed and transferred into the O2k chamber set at 40°C and containing 1 ml of Mir05. We first recorded mitochondrial *ROUTINE* respiration representing O_2_ consumption with endogenous substrates and adenosine diphosphate (ADP). Subsequently, we added 5 µl of 2 mM pyruvate and then inhibited ATP synthase with 2.5 μl of 0.5 mM oligomycin to record *LEAK* respiration, corresponding to O_2_ consumption not linked to ATP synthesis. OXPHOS (oxidative phosphorylation) respiration linked to ATP synthesis was then calculated by subtracting *LEAK* from *ROUTINE* values. We then step-titrated 1 μl of 1 mM CCCP (carbonyl cyanide m-chlorophenyl hydrazine) until reaching maximum O_2_ consumption limited only by the electron transport system (ETS respiration) and potentially by the availability of endogenous substrate. Finally, mitochondrial respiration was inhibited with 5 μl of 1 mM of antimycin A to quantify non-mitochondrial O_2_ consumption and used this as a correction factor for the other respiration rates measured (see electronic supplementary material, figure S1 for a representative Oroboros trace). Blood cells contained in the sample were counted with an automatic cell counter (TC20 by Bio-Rad), and this amount was used to statistically control for potential differences in the amount of blood cells between individuals.

We calculated two flux control ratios (FCR) from respirations rates, namely: (i) *OXPHOS coupling efficiency* (*E*) that reflects the efficiency of mitochondria to use O_2_ to produce ATP, calculated as 1 − (*LEAK* / *ROUTINE*). This ratio ranges from 0 to 1 and represents the proportion of O_2_ linked to ATP production (e.g. a ratio of 0.80 meaning that 80% of O_2_ is used to produce ATP, 20% being ‘lost’ mainly to compensate proton leak). (ii) *OXPHOS reserve capacity* (FCR = 1 − (*ROUTINE* / *ETS*)) representing the scope to increase respiration from endogenous conditions to maximum respiration; a ratio of 0.80 indicating that respiration can increase by 80% compared with endogenous conditions.

### (d) Statistical analysis

During preliminary analyses, we first ran a linear model to make sure our two dependent variables (take-off energy and flight endurance) were not correlated to each other (*p* = 0.91), and thus we treated them as independent. We fitted linear models on take-off energy and flight endurance, with mitochondrial aerobic metabolism parameters, body mass (due to its known effect on flight [22]) and blood cell count (due to the known effect of haematocrit on physical performance [34]) as predictors. Since we found strong correlations among the majority of the mitochondrial predictors, we tested them in separate models to avoid multicollinearity. Model assumptions and outlier presence were checked using DHARMa package [35], and no violations of assumptions were detected. Blood cell count was never significantly correlated to mitochondrial aerobic metabolism, so respiration rates were expressed as pmol O_2_ s^−1^ μl blood^−1^ [33]. Model estimates are presented with standard errors. All analyses were performed with R v. 4.1.2 for Mac [36].

## 3. Results

**Figure 1.**
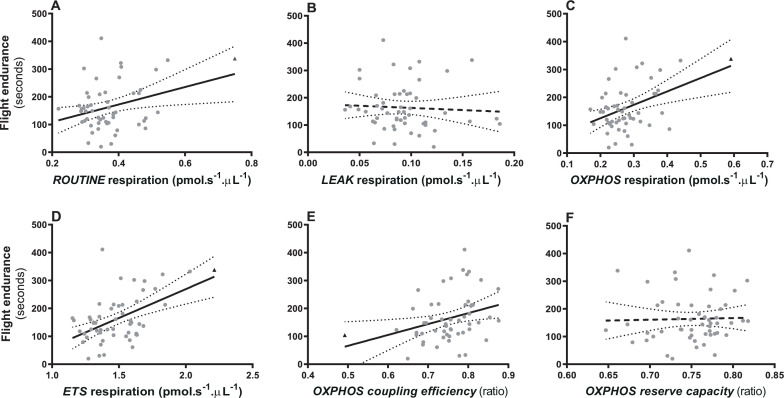
Relationships between flight endurance and blood cells mitochondrial respiration rates under endogenous conditions (*ROUTINE*: (A)), ATP-synthesis inhibition (*LEAK*: (B)), restricted to ATP production (*OXPHOS*: (C)), maximum stimulation (*ETS*: (D)), *OXPHOS* coupling efficiency (E), *OXPHOS* reserve capacity (FCR). Non-significant relationships are plotted with dashed black lines. Ninety-five per cent confidence intervals are presented. One potentially influential data point is presented with a black triangle (see text).

Flight endurance to exhaustion ranged between 20 and 411 s (mean ± s.d. = 163.8 ± 85.39) and was positively correlated with mitochondrial respiration rates under *ROUTINE* (*b* = 5.780 ± 2.739, *F* = 4.452, *p* = 0.040, *R*^2^ = 0.16, figure 1A), *OXPHOS* (*b* = 8.919 ± 3.148, *F* = 8.311, *p* = 0.006, *R*^2^ = 0.22, figure 1C) and *ETS* (*b* = 3.858 ± 1.040, *F* = 13.764, *p* < 0.001, *R*^2^ = 0.29, figure 1D) conditions (figure 1A–C) but not *LEAK* (*b* = −3.837 ± 7.542, *F* = 0.259, *p* = 0.613, *R*^2^ = 0.09, figure 1B). The removal of one influential data point (see figure 1) weakened these relationships, becoming non-significant for *ROUTINE* (*p* = 0.27), marginally significant for *OXPHOS* (*p* = 0.057) but remaining highly significant for *ETS* (*p* = 0.005). Flight endurance to exhaustion was positively correlated with *OXPHOS* coupling efficiency (*β* = 345.097 ± 162.737, *F* = 4.452, *p* = 0.038, *R*^2^ = 0.16; figure 1E) but not with reserve capacity (*β* = 74.533 ± 306.679, *F* = 0.059, *p* = 0.809, *R*^2^ = 0.09; figure 1F). These two dependent variables were positively related to body mass (*β* = 13.542 ± 6.701, *F* = 4.084, *p* = 0.048) and (*β* = 15.583 ± 6.927, *F* = 5.062, *p* = 0.028), respectively. Removing one potential influential point for *OXPHOS* coupling efficiency (figure 1E) did not change the relationship between that and flight endurance to exhaustion (*p* = 0.045). Body mass was positively correlated with flight endurance but only in the model including *LEAK* respiration (*β* = 15.610 ± 7.130, *F* = 4.791, *p* = 0.033, *R*^2^ = 0.09; 0.14 < *p* < 0.33 in the three other models). Blood cell count showed no significant correlation with flight endurance (all *p* > 0.39).

**Figure 2.**
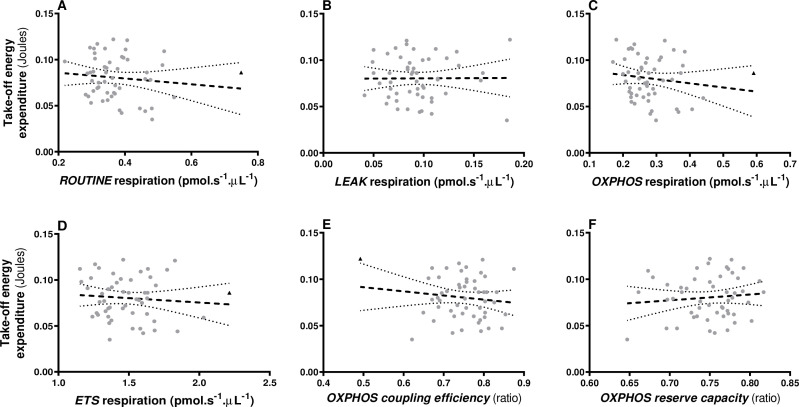
Relationships between take-off energy expenditure (A–F) and blood cells mitochondrial respiration rates under endogenous conditions (*ROUTINE*: A), ATP-synthesis inhibition (*LEAK*: B), restricted to ATP production (*OXPHOS*: C), maximum stimulation (*ETS*: D), *OXPHOS* coupling efficiency (E), *OXPHOS* reserve capacity (FCR). Non-significant relationships are plotted with dashed black lines. Ninety-five per cent confidence intervals are presented. One potentially influential data point is presented with a black triangle (see text).

Take-off energy expenditure was not significantly correlated to mitochondrial respiration rates, *OXPHOS* coupling efficiency or reserve capacity (*F* < 0.78, all *p* > 0.44, figure 2) and showed no significant relationship with body mass nor with blood cell count (all *p* > 0.28, see electronic supplementary material, table S1).

## 4. Discussion

Here, we demonstrate that mitochondrial aerobic metabolism measured in blood cells of house sparrows appears to be a meaningful metric correlated to organismal aerobic endurance. We examined several correlated mitochondrial measures, and the results of each are not independent of the others. Nevertheless, the OXPHOS and ETS respiratory states are most closely associated with cellular energy production in the form of ATP, and it makes sense that they are positively related to a sparrow’s ability to endure flight for a sustained period, as ATP provides the fuel for flight muscles. Mitochondrial respiration rates are influenced by mitochondrial density (e.g. [37]). While we did not assess mitochondrial density, the fact that LEAK respiration did not correlate with flight endurance suggests that variation in mitochondrial density is not the main driver of the relationships we observed for OXPHOS and ETS. Importantly, OXPHOS coupling efficiency, which denotes the ability of an organism to meet cellular energy demands by converting a higher proportion of oxygen into ATP and by producing less heat, showed a positive relationship with endurance. This is an important result as OXPHOS coupling efficiency is an a-dimensional ratio independent from variation in mitochondrial density and is expected to impact exercise and locomotion efficiency [38,39].

Our results, based on the activity of mitochondria in blood cells, agree with another previous observational study supporting a relationship between locomotion and mitochondrial aerobic metabolism in pectoral muscles [40]. While we pushed our birds to exhaustion by actively chasing them, our study was not experimental and therefore prevents us from drawing inferences about any causal link. Indeed, the possibility remains that instead of mitochondrial aerobic metabolism determining flight endurance, the opposite could be true, with flight endurance being driven by other variables such as feather quality, age differences, muscle conditions and health status [32]. Future tests manipulating mitochondrial coupling efficiency [41] are needed to establish a solid causal link with flight endurance, as found for swimming capacity in fish [42]. Additionally, as we only used females, future studies should also include males in their measurements, similarly to a recent study on king penguins demonstrating sex-specific differences in blood cell aerobic metabolism [43].

While we had a more sophisticated metric to assess total energy in take-off compared with our relatively coarse endurance test, none of our measurements of mitochondrial metabolism were correlated with it. Given that take-off energy is an anaerobic phase of locomotion that does not require the use of mitochondrial oxidative processes, this was expected. However, this negative result remains an important test that indirectly supports the quality of our mitochondrial measurements and further strengthens our findings related to the importance of mitochondrial output during the aerobic phase of flight.

It is an important advance that blood cell mitochondrial metabolism correlates with one metric of flight performance at the whole organismal level and raises the question of what may be the underlying mechanisms that may explain this relationship. Red blood cells, despite being the most abundant cell type in the avian and mammalian body alike [44], make a minimal contribution to the overall energy turnover of the organism compared with more energetically active organs such as brain, liver and heart [45]. In particular, flight in birds is almost entirely powered by pectoral muscles [46], which were indeed the tissue used to investigate mitochondrial correlates of locomotion in previous studies [38,40]. Since flight endurance is linked to muscle aerobic metabolism, then blood cell and muscle aerobic metabolism may be correlated with each other as shown previously in the king penguin [12]. While a role of ATP produced by avian blood cells’ mitochondria in directly powering the energetic costs of flight seems unlikely, recent works suggest that erythrocytes may perform a set of functions similar to those of an organ [47]. Indeed, studies in humans show that intravascular release of ATP from erythrocytes plays an important part in orchestrating oxygen distribution to organs actively involved in exercise [48,49], and ADP/ATP ratio influences Hb-O_2_ affinity, including in nucleated red blood cells [50]. While this explanation awaits validation in avian systems, it also provides a plausible mechanism describing how, despite minimal ATP production in comparison with other tissues, blood mitochondria may reflect the metabolic performances of other organs and whole body [15,17].

Mitochondrial assays can be run on blood cells with minimal impact on animal welfare, avoiding the need for terminal sampling or invasive biopsies from animal tissues. This less intrusive approach can effectively complement the established and detailed insights gained from studies on muscle tissues, offering a more accessible window into a series of interesting possibilities for future investigations. Longitudinal sampling of blood during postnatal growth can help us elucidate metabolic development during the ontogeny of flight. Additionally, blood can be ideal for broad comparative tests that require large sample sizes. Interspecific studies exploring relationships between mitochondrial variation and flight capacities may greatly benefit our understanding of evolutionary differences in migratory strategies [40]. Ultimately, our results suggest that the use of blood cells may be a valid and minimally invasive tool for ecophysiological studies of non-mammalian vertebrates, all of which have nuclei and active mitochondria [11]. This offers significant ethical and technical advantages for advancing our understanding of the links between mitochondrial physiology and ecologically relevant traits.

## Data Availability

Data can be accessed from the Dryad Digital Repository [51]. Supplementary material is available online [52].
